# Collagen binding specificity of the discoidin domain receptors: Binding sites on collagens II and III and molecular determinants for collagen IV recognition by DDR1

**DOI:** 10.1016/j.matbio.2010.10.004

**Published:** 2011-01

**Authors:** Huifang Xu, Nicolas Raynal, Stavros Stathopoulos, Johanna Myllyharju, Richard W. Farndale, Birgit Leitinger

**Affiliations:** a National Heart and Lung Institute, Imperial College London, London SW7 2AZ, United Kingdom; b Department of Biochemistry, University of Cambridge, Cambridge CB2 1QW, United Kingdom; c Oulu Centre for Cell-Matrix Research, Biocenter Oulu, and Department of Medical Biochemistry and Molecular Biology, University of Oulu, Oulu FIN-90014, Finland

**Keywords:** DDR, discoidin domain receptor, DS, discoidin homology, HEK, human embryonic kidney, RTK, receptor tyrosine kinase, VWF, von Willebrand factor, Collagen receptor, Discoidin domain receptor, Receptor tyrosine kinase, Cell–extracellular matrix interaction, Collagen binding specificity

## Abstract

The discoidin domain receptors, DDR1 and DDR2 are cell surface receptor tyrosine kinases that are activated by triple-helical collagen. While normal DDR signalling regulates fundamental cellular processes, aberrant DDR signalling is associated with several human diseases. We previously identified GVMGFO (O is hydroxyproline) as a major DDR2 binding site in collagens I–III, and located two additional DDR2 binding sites in collagen II. Here we extend these studies to the homologous DDR1 and the identification of DDR binding sites on collagen III. Using sets of overlapping triple-helical peptides, the Collagen II and Collagen III Toolkits, we located several DDR2 binding sites on both collagens. The interaction of DDR1 with Toolkit peptides was more restricted, with DDR1 mainly binding to peptides containing the GVMGFO motif. Triple-helical peptides containing the GVMGFO motif induced DDR1 transmembrane signalling, and DDR1 binding and receptor activation occurred with the same amino acid requirements as previously defined for DDR2. While both DDRs exhibit the same specificity for binding the GVMGFO motif, which is present only in fibrillar collagens, the two receptors display distinct preferences for certain non-fibrillar collagens, with the basement membrane collagen IV being exclusively recognised by DDR1. Based on our recent crystal structure of a DDR2-collagen complex, we designed mutations to identify the molecular determinants for DDR1 binding to collagen IV. By replacing five amino acids in DDR2 with the corresponding DDR1 residues we were able to create a DDR2 construct that could function as a collagen IV receptor.

## 1 Introduction

Collagens constitute the most abundant protein family in vertebrates and are crucial for the development, growth, and mechanical stability of connective tissues (Myllyharju and Kivirikko, 2004). Collagens are characterised by the presence of triple-helical domains, composed of three collagen α chains with repeated Gly-X-X′ motifs, where X is frequently proline and X′ 4-hydroxyproline (Hyp, O) (Brodsky and Persikov, 2005). Most collagens self-assemble into higher order structures such as fibrils or networks. The fibrillar collagens (types I, II, III, V, and XI) are mainly constituted of uninterrupted triple-helical domains of ~ 1000 amino acids and play important architectural roles in many connective tissues, while the network-forming collagen type IV is a key component of basement membranes. In addition to determining mechanical properties of tissues, collagens also interact with other extracellular matrix proteins and cell surface receptors, thus regulating fundamental cellular processes such as adhesion and signalling (Heino, 2007; Leitinger and Hohenester, 2007).

The most widely expressed mammalian receptors for collagen are four members of the β1 integrin family and two homologous receptor tyrosine kinases (RTKs), the discoidin domain receptors DDR1 and DDR2. The interactions of collagens with integrins have been extensively characterised (Leitinger and Hohenester, 2007), in particular for α1β1 and α2β1, and are understood in atomic detail for the α2β1 integrin (Emsley et al., 2000). Integrins recognise discrete amino acid sequences in triple-helical collagen, such as the high-affinity binding motif GFOGER (Knight et al., 2000; Zhang et al., 2003). The interactions of the DDRs with collagen are less well understood. Both DDRs interact with several collagen types in their native triple-helical conformation (Shrivastava et al., 1997; Vogel et al., 1997). Collagen-induced DDR signalling controls cell proliferation, adhesion and migration, as well as re-modelling of extracellular matrices (reviewed in Vogel et al., 2006). Both receptors play important roles in development. DDR1 controls mammary gland development (Vogel et al., 2001) and kidney function (Gross et al., 2004), while DDR2 regulates the growth of long bones (Ali et al., 2010; Bargal et al., 2009; Kano et al., 2008; Labrador et al., 2001). Aberrant DDR function is associated with disease progression in fibrotic disorders of the lung, liver and kidney, atherosclerosis, osteoarthritis, rheumatoid arthritis and several types of cancer (Vogel et al., 2006).

The DDRs are composed of an extracellular region, a transmembrane domain, a large cytosolic juxtamembrane domain, and a C-terminal tyrosine kinase domain. Unlike canonical RTKs, which are thought to undergo ligand-induced dimerisation, the DDRs are pre-dimerised at the cell membrane in the absence of collagen (Abdulhussein et al., 2008; Mihai et al., 2009; Noordeen et al., 2006). The DDR extracellular region consists of two domains: an N-terminal discoidin homology (DS) domain and a so-called stalk region unique to DDRs. Collagen binds to a specific site in the DS domain (Abdulhussein et al., 2004; Ichikawa et al., 2007; Leitinger, 2003), which results in slow and sustained autophosphorylation of cytoplasmic tyrosine residues (Shrivastava et al., 1997; Vogel et al., 1997).

Identifying interaction motifs for proteins on fibrillar collagens is made difficult by the large size of the collagenous domains and repetitive nature of the Gly-X-X′ motif. The use of synthetic triple-helical peptide analogues of collagen has allowed the identification of specific amino acid motifs within collagen that function as binding sites for interacting proteins (e.g. Knight et al., 2000; Zhang et al., 2003). Moreover, our recent development of the Collagen Toolkits has made possible the comprehensive analysis of binding sites within fibrillar collagen types II and III (Farndale et al., 2008). The Collagen Toolkits are sets of overlapping triple-helical peptides encompassing the entire triple-helical domains of collagens II and III (Konitsiotis et al., 2008; Raynal et al., 2006). In a previous study we used the Collagen II Toolkit to identify binding sites for DDR2 (Konitsiotis et al., 2008). Using truncated and alanine-substituted peptides, we defined a GVMGFO motif as a major DDR2-binding site present in collagens I–III. Our identification of the GVMGFO motif as a DDR2 ligand enabled the determination of a crystal structure of a complex between the DDR2 DS domain and a collagen peptide (Carafoli et al., 2009).

While binding sites for DDR2 on collagen II have been mapped (Konitsiotis et al., 2008), a similar analysis on collagen III has not been performed. Furthermore, the specific DDR1 binding sites on collagen are unknown. In the present work we extend our previous study to the analysis of DDR1 and DDR2 binding sites on collagens II and III. For DDR2, several distinct binding sites were defined on both collagens, whereas the interaction of DDR1 with the collagen peptides was mostly restricted to the GVMGFO motif. DDR1 binding and induction of receptor autophosphorylation occurred with the same amino acid requirements as previously defined for DDR2. Thus, both DDRs interact with the same specificity with the GVMGFO motif, which is found in the fibrillar collagens I–III (Konitsiotis et al., 2008). In contrast, the two receptors are known to have distinct preferences for certain non-fibrillar collagen types, such as the exclusive binding of DDR1 to the basement membrane collagen IV (Shrivastava et al., 1997; Vogel et al., 1997), and the preferred binding of DDR2 to collagen X (Leitinger and Kwan, 2006). Based on our recent DDR2-collagen peptide crystal structure (Carafoli et al., 2009), we here define the molecular determinants for the different collagen IV-binding specificities of the two DDRs. By replacing five amino acids in DDR2 with DDR1 residues we were able to create a DDR2 construct that could function as a collagen IV receptor.

## 2 Results

### 2.1 New recombinant proteins to analyse DDR collagen binding

**Fig. 1:**
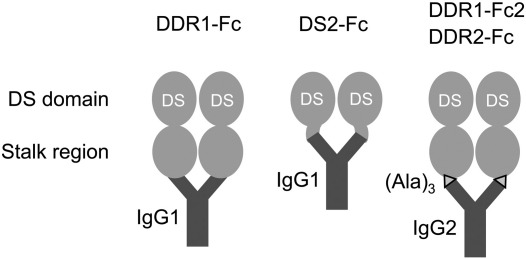
Schematic representation of recombinant DDR-Fc constructs. The DDR extracellular domains contain an N-terminal DS domain that contains the collagen binding site, followed by a so-called stalk region. Fc-tagged proteins are fused to the Fc fragment of human IgG1 or IgG2, as indicated, with the Fc sequences (and linker amino acids) added after the last amino acid of the DDR ectodomains (T416 for DDR1, T398 for DDR2). The new constructs are shown on the right, and contain a three amino acid linker (Ala-Ala-Ala), symbolized by a triangle, between the DDR ectodomains and the IgG2 Fc sequence.

We previously characterised binding of DDR extracellular domain constructs to collagen using solid phase binding assays. Both DDRs showed high-affinity binding to collagen I (Leitinger, 2003) but differed in their preference for collagens II, IV and X (Leitinger, 2003; Leitinger and Kwan, 2006; Leitinger et al., 2004). Our previous studies used two types of recombinant DDR ectodomain constructs: DDR1-Fc, comprising the entire DDR1 ectodomain, tagged with a dimerising human Fc tag (IgG1 sequence; see Fig. 1); and His-DDR2, comprising the DDR2 ectodomain tagged with a His tag and a Myc epitope (Leitinger, 2003). While constructs with both types of tags had been created for DDR1 and DDR2, only His-DDR2, but not His-DDR1, was functional. Conversely, only DDR1-Fc, but not DDR2-Fc, could be obtained, as the respective DDR2-Fc construct was not secreted from transfected cells. However, for DDR2, another Fc construct was successfully obtained: DS2-Fc, mainly consisting of the DDR2 DS domain, C-terminally tagged with human IgG1. DS2-Fc showed the same collagen binding specificity for fibrillar collagens as His-DDR2 (Konitsiotis et al., 2008; Leitinger, 2003; Leitinger and Kwan, 2006; Leitinger et al., 2004).

To compare the collagen binding specificities of DDR1 and DDR2 independently of the added tag, we have now created a new set of DDR ectodomain constructs, fused to the same Fc tag. By using an expression vector that introduces a spacer between the DDR ectodomains and the Fc tag (IgG2 sequence; see Methods), we successfully obtained DDR2-Fc, and an equivalent DDR1 construct, which we term DDR1-Fc2 (Fig. 1). The new DDR-Fc proteins showed the same collagen binding specificities as the respective earlier ectodomain constructs, with DDR1-Fc2 binding well to collagens I and IV and DDR2-Fc exhibiting high-affinity binding to collagens I and II, but no binding to collagen IV (data not shown and Fig. 8), demonstrating that the tags do not influence the ability of the DDR ectodomains to bind collagen. We have used the new DDR-Fc constructs to extend our analysis of DDR binding sites on collagen.

### 2.2 DDR2 binds to more sites on human collagens II and III than DDR1

**Fig. 2:**
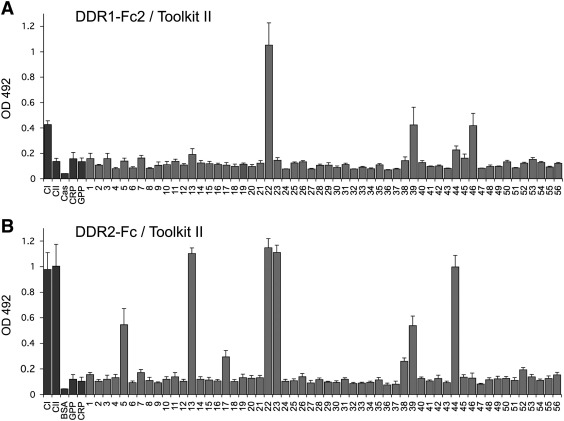
Identification of DDR1 and DDR2 binding sites on collagen II. Binding of recombinant DDR1-Fc2 or DDR2-Fc to immobilized collagen II Toolkit peptides in a solid phase binding assay. Recombinant DDR-Fc proteins were added for 3 h at room temperature to 96 wells coated with collagen or peptides at 10 μg/ml. Bound proteins were detected with anti-Fc antibodies and measured as absorbance at 492 nm. (A) Binding of DDR1-Fc2, added at 20 μg/ml (220 nM). (B) Binding of DDR2-Fc, added at 10 μg/ml (110 nM). Shown are the mean +/− SD of three to four independent experiments, each performed in triplicates. CI: rat tail collagen I; CII: bovine collagen II; Cas: casein; GPP and CRP: peptides without specific collagen sequences. GPP: (GPP)_10_; CRP: (GPO)_10_.

Our previous study identified DDR2 binding sites on collagen II (Konitsiotis et al., 2008), but did not include any data on DDR1 binding sites, or on DDR binding sites on human collagen III. To identify sites in human collagens that act as DDR1 or DDR2 binding sites, we used the Collagen II and Collagen III Toolkits, two sets of overlapping synthetic triple-helical peptides encompassing the entire triple-helical domains of collagens II and III, respectively (Konitsiotis et al., 2008; Raynal et al., 2006). Recombinant DDRs were used to screen the Toolkit peptides for DDR binding in solid phase binding assays. Only one Collagen II Toolkit peptide, II-22, showed high binding to DDR1-Fc2, while peptides II-39 and II-46 gave a more modest response (Fig. 2A). A similar result was obtained using DDR1-Fc (Supplementary Fig. 1). In contrast to DDR1-Fc, DDR2-Fc showed high binding to several peptides (Fig. 2B), as expected from our previous results with His-DDR2 and DS2-Fc, which had identified three main DDR2 binding sites, resulting from DDR2 binding to Toolkit peptides II-13, II-22, II-23 and II-44 (Konitsiotis et al., 2008). In the present study, very similar results were obtained for DDR2-Fc, with strong binding to peptides II-13, II-22, II-23 and II-44 (Fig. 2B). Additionally, significant DDR2-Fc binding was also seen for peptides II-5 and II-39.

**Fig. 3:**
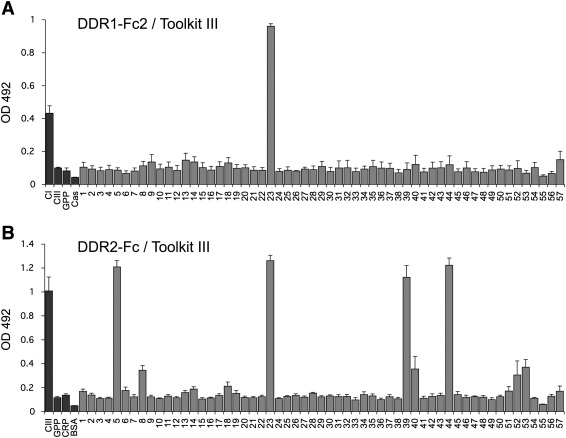
Identification of DDR1 and DDR2 binding sites on collagen III. Binding of recombinant DDR1-Fc2 or DDR2-Fc to immobilized collagen III Toolkit peptides in a solid phase binding assay. Recombinant DDR-Fc proteins were added for 3 h at room temperature to 96 wells coated with collagen or peptides at 10 μg/ml. Bound proteins were detected with anti-Fc antibodies and measured as absorbance at 492 nm. (A) Binding of DDR1-Fc2, added at 20 μg/ml (220 nM). (B) Binding of DDR2-Fc, added at 10 μg/ml (110 nM). Shown are the mean +/− SD of three to four independent experiments, each performed in triplicates. CI: rat tail collagen I; CIII: recombinant human collagen III; Cas: casein; GPP and CRP peptides as in Fig. 2.

The binding assays conducted with the Collagen III Toolkit showed very restricted binding of DDR1, with only one peptide, III-23, interacting with DDR1-Fc and DDR1-Fc2 (Fig. 3A and Supplementary Fig. 2A). In contrast, several peptides interacted with DDR2. In addition to binding to III-23, DDR2-Fc bound III-5, III-39 and III-44 (Fig. 3B). His-DDR2 (data not shown) and DS2-Fc displayed the same binding specificities as DDR2-Fc (Supplementary Fig. 2B).

### 2.3 DDR1 and DDR2 bind the GVMGFO motif with the same amino acid requirements

**Fig. 4:**
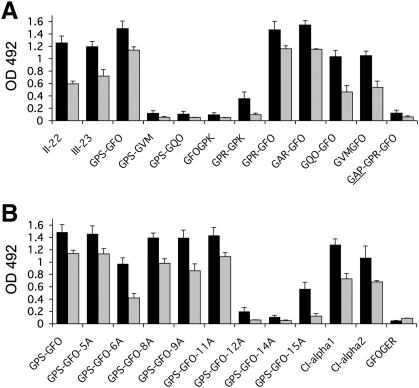
Characterisation of collagen sequence required for DDR1 binding. Recombinant DDR1-Fc protein was added for 3 h at room temperature to 96 wells coated with collagen-derived peptides or collagen at 10 μg/ml. (A) Binding of DDR1-Fc to collagen III Toolkit peptide, III-23, and truncated peptides derived from this sequence (Table 1). (B) Binding of DDR1-Fc to alanine-substituted peptides derived from collagen III Toolkit peptide, III-23 (Table 1). Shown are the mean +/− SD of four independent experiments, each performed in triplicates. Black bars, DDR1-Fc added at 15 μg/ml (165 nM); grey bars, DDR1-Fc added at 3 μg/ml (33 nM).

The overlapping sequence of peptides II-22 and II-23 is related to the central guest sequence of III-23 (Table 1). In our previous work we identified the sequence GVMGFO, contained in these peptides, as the minimum collagen sequence required for DDR2 binding (Konitsiotis et al., 2008). Using a set of truncated peptides derived from III-23 (Table 1), we obtained a similar result for DDR1 (Fig. 4A). With the exception of the peptide designated as GPR-GPK (see Table 1), DDR1Fc bound strongly to peptides containing the intact GVMGFO sequence, but did not bind to any peptides in which this hexapeptide sequence was truncated. DDR binding to intact collagen is strictly dependent on the native triple-helical collagen conformation. Similar to DDR2 (Konitsiotis et al., 2008), DDR1-Fc did not interact with peptide GAP-GPR-GFO, a non-helical peptide version of the high-affinity DDR ligand with guest sequence GPRGQOGVMGFO (Fig. 4A, last lanes). This indicates that peptide recognition by DDR1 in our system mirrors the interaction of DDR1 with native collagen. Furthermore, like DDR2 (Konitsiotis et al., 2008), DDR1 did not bind to a peptide containing the guest sequence GFOGER, a high-affinity integrin binding sequence (Knight et al., 2000) (Fig. 4B).

DDR1-Fc bound a set of alanine-substituted peptides (Table 1) with the same specificity as we previously observed for DDR2 (Konitsiotis et al., 2008) (Fig. 4B). Substituting either Phe or Met for Ala in GVMGFO was detrimental for DDR1 binding, while substituting Hyp for Ala in GVMGFO had a more modest effect. Replacing Arg in GPRGQOGVMGFO led to a modest reduction in binding, while all other X and X′ positions could be substituted without apparent loss of DDR1 binding (Fig. 4B).

In the heterotrimeric collagen I, composed of two α1 chains and one α2 chain, the human sequence related to GPRGQOGVMGFO in collagen III and GARGQOGVMGFO in collagen II is GARGQ*A*GVMGFO, which differs in two amino acids (underlined) from the collagen III sequence and one amino acid (underlined and italic) from the collagen II sequence. This sequence is found in the α1 chain. As expected from the results of the alanine-substituted peptides, this guest sequence interacted well with DDR1 (Fig. 4B). The sequence of the α2 chain that aligns with this sequence, GARGEOGNIGFO (where underlining highlights difference to the motif GARGQOGVMGFO in collagen II), was also recognised by DDR1 with similar apparent affinity, indicating that both collagen α chains may contribute to the corresponding DDR1 binding site in collagen I.

### 2.4 Activation of DDR1 transmembrane signalling by GVMGFO-containing peptides

**Fig. 5:**
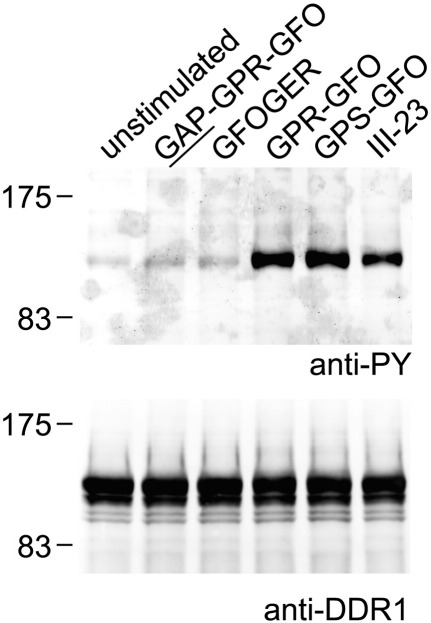
DDR1 binding peptides mediate autophosphorylation of cell surface DDR1. Full length DDR1a was transiently expressed in HEK293 cells. After stimulation for 90 min with collagen peptides at 100 μg/ml, cell lysates were analysed by SDS-PAGE and Western blotting. Peptide names refer to Table 1. The blot was probed with anti-phosphotyrosine mAb 4G10 (upper panel), followed by stripping and reprobing with anti-DDR1 (lower panel). The positions of molecular weight markers (in kDa) are indicated. The experiment was carried out four times with very similar results.

We previously showed that DDR2 activation, as measured by receptor autophosphorylation, could be induced by incubating cells expressing full-length DDR2 with triple-helical peptides containing high-affinity GVMGFO motifs (Konitsiotis et al., 2008). Similar results were obtained in the present study for DDR1. Strong DDR1 autophosphorylation was induced with peptides containing GVMGFO in triple-helical form (peptides III-23, GPR-GFO and GPS-GFO, see Table 1) but not in non-helical form (compare GAP-GPR-GFO to GPR-GFO) (Fig. 5). As previously established for DDR2, DDR1 was not activated by the non-binding peptide GPS-GFO-14A, in which Phe in GVMGFO is replaced by Ala, while other substitutions that retained DDR1 binding induced receptor autophosphorylation (Supplementary Fig. 3). Moreover, the integrin ligand GFOGER could not induce DDR1 phosphorylation (Fig. 5). Thus, triple-helical peptides comprising the DDR binding site activate DDR1 autophosphorylation in a specific manner.

### 2.5 Only GVMGFO-containing peptides activate DDR2 transmembrane signalling

**Fig. 6:**
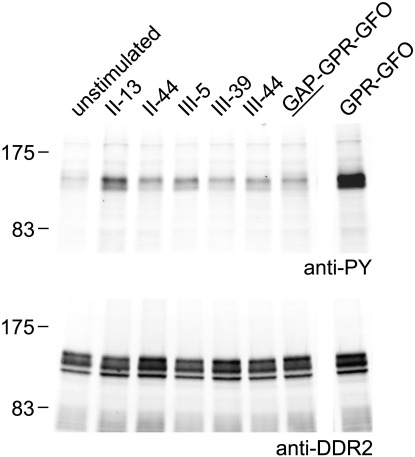
DDR2 binding peptides that do not contain the GVMGFO motif do not mediate strong autophosphorylation of cell surface DDR2. Full length DDR2 was transiently expressed in HEK293 cells. After stimulation for 90 min with collagen peptides, cell lysates were analysed by SDS-PAGE and Western blotting. Peptide names refer to Table 1. Peptides were used at 500 μg/ml, except peptide GPR-GFO, which was used at 100 μg/ml. Cell lysates were resolved on two gels. The corresponding blots were probed with anti-phosphotyrosine mAb 4G10 (upper panel) or anti-DDR2 (lower panel). The positions of molecular weight markers (in kDa) are indicated.

As shown in Figs. 2B and 3B, DDR2 displayed strong binding to a number of peptides in addition to the GVMGFO-containing peptides II-22, II-23 and III-23. We tested the functional effects on DDR2 autophosphorylation of the peptides II-13, II-44, III-5, III-39 and III-44 (Fig. 6). Strong phosphorylation of DDR2, comparable to that induced by collagen I, can be mediated by GVMGFO-containing peptides when used at 50–100 μg/ml in the medium (Konitsiotis et al., 2008). In contrast to GVMGFO-containing peptides, none of the additional DDR2 binding peptides induced DDR2 autophosphorylation when tested at 100–300 μg/ml (data not shown), but peptide II-13 induced a weak phosphorylation signal when the peptide concentration was increased to 500 μg/ml (Fig. 6). Thus, only GVMGFO-containing peptides are able to act as receptor agonists, inducing the full DDR2 phosphorylation signal.

### 2.6 DDR1/DDR2 hybrid proteins to analyse determinants for difference in DDR collagen binding specificity

**Fig. 7:**
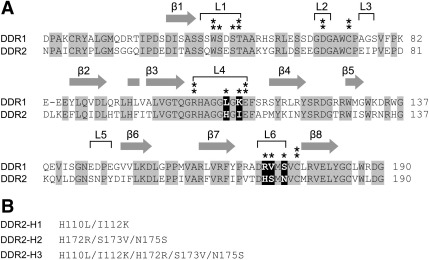
Sequence conservation of the DDR collagen binding sites. (A) Sequence alignment of the DS domains of human DDR1 and DDR2. Secondary structure elements are given above the alignment, sequence numbering to the right of the alignment. Conserved residues are highlighted in grey. The central residues of the GVMGFO binding interface (Trp52, Thr56, Asp69, Arg105, Glu113, and Cys73-Cys177 disulfide bridge (see Carafoli et al., 2009)), are indicated by two asterisks. Additional residues that lose ≥ 5 Å^2^ of their solvent-accessible surface upon collagen binding (see Carafoli et al., 2009) are indicated by one asterisk. Non-conserved residues that lose ≥ 5 Å^2^ of their solvent-accessible surface upon collagen binding are highlighted in white on black background. (B) Amino acid replacements in DDR2–DDR1 hybrid constructs.

**Fig. 8:**
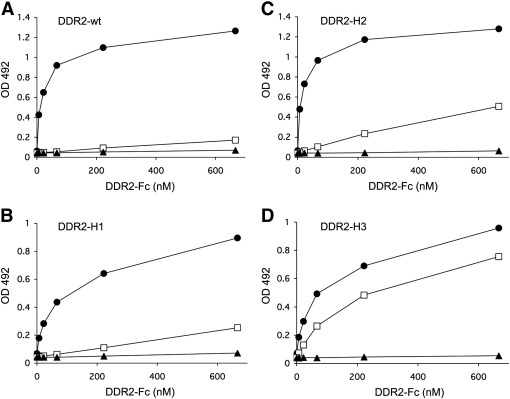
Replacing five amino acids in the collagen binding site of DDR2 with DDR1 amino acids leads to collagen IV recognition. Solid phase binding assay with recombinant DDR2 wild-type and DDR2–DDR1 hybrid constructs. DDR2-Fc proteins were added for 3 h at room temperature to 96 wells coated with collagen at 10 μg/ml. Bound proteins were detected with anti-Fc antibodies and measured as absorbance at 492 nm. (A) DDR2 wild-type, (B) DDR2-H1, (C) DDR2-H2, (D) DDR2-H3 binding to different immobilized proteins: BSA (▲); human placental collagen IV (□); rat tail collagen I (●). Shown is a representative of three independent experiments, each performed in duplicates.

The data so far show that DDR1 and DDR2 bind fibrillar collagens in a similar manner. However, DDR1 and DDR2 differ in their specificity for binding to the non-fibrillar basement membrane collagen IV. We recently obtained a crystal structure of a complex between the DDR2 DS domain and a triple-helical collagen peptide encompassing the GVMGFO motif (Carafoli et al., 2009). The main collagen binding residues defined by this structure (Trp52, Thr56, Asp69, Arg105, Glu113, and Cys73-Cys177 disulfide bridge) are strictly conserved in the homologous DDR1 (Fig. 7), consistent with the binding of both DDRs to GVMGFO-containing peptides, as shown in Figs. 2–4. We previously noted that several DDR residues at the periphery of the GVMGFO peptide-binding interface are not conserved, and speculated that these residues may be responsible for the distinct collagen binding specificity of the two DDRs (Carafoli et al., 2009). In order to test this hypothesis, we created DDR2–DDR1 hybrid constructs, in which these non-conserved amino acid residues were replaced in DDR2 with the corresponding DDR1 amino acids (Fig. 7). Three DDR2 constructs were obtained: one in which two residues in loop 4 of DDR2 were replaced by DDR1 amino acids (DDR2-H1), one in which three loop 6 residues were replaced by corresponding DDR1 residues (DDR2-H2), and a third construct that combined the mutations of the former constructs (DDR2-H3). All three constructs were made as the DDR2-Fc versions and tested for their ability to bind to collagen IV. Wild-type DDR2-Fc, as expected, showed no collagen IV binding (Fig. 8A). The two hybrid constructs DDR2-H1 and DDR2-H2 only showed minimal collagen IV binding (Fig. 8B and C), indicating that replacing DDR2 amino acids in individual loops 4 or 6 is not sufficient for switching collagen binding specificity. DDR2-H3, on the other hand, showed robust binding to collagen IV (Fig. 8D).

**Fig. 9:**
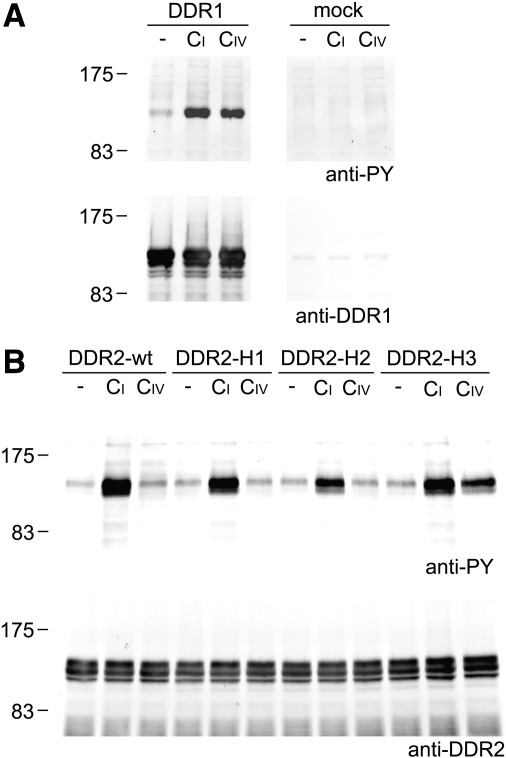
Replacing five amino acids in the collagen binding site of DDR2 with DDR1 amino acids leads to collagen IV-induced signalling of cell surface DDR2-H3. Full length DDR1a, DDR2 wild-type or DDR2-DDR1 hybrid constructs were transiently expressed in HEK293 cells. After stimulation for 90 min with collagen at 10 μg/ml (collagen I, *CI*) or 50 μg/ml (collagen IV, *CIV*), cell lysates were analysed by SDS-PAGE and Western blotting. (A) DDR1 expression. The blot was probed with anti-phosphotyrosine mAb 4G10 (upper panel), followed by stripping and reprobing with anti-DDR1 (lower panel). (B) DDR2 wild-type and DDR2-DDR1 hybrid receptor expression. Cell lysates were resolved on two gels. The corresponding blots were probed with anti-phosphotyrosine mAb 4G10 (upper panel) or anti-DDR2 (lower panel). The positions of molecular weight markers (in kDa) are indicated. The experiment was carried out three times with very similar results.

We also introduced the DDR1 amino acids in the context of full-length DDR2, and expressed these hybrid receptors in HEK293 cells. Similar to the binding assays, receptor activation assays showed that DDR2-H3, but not DDR2-H1 or DDR2-H2 could be activated by collagen IV (Fig. 9). Together, these results demonstrate that DDR residues outside the GVMGFO collagen binding pocket determine the specificity for collagen IV of the two DDRs.

**Fig. 10:**
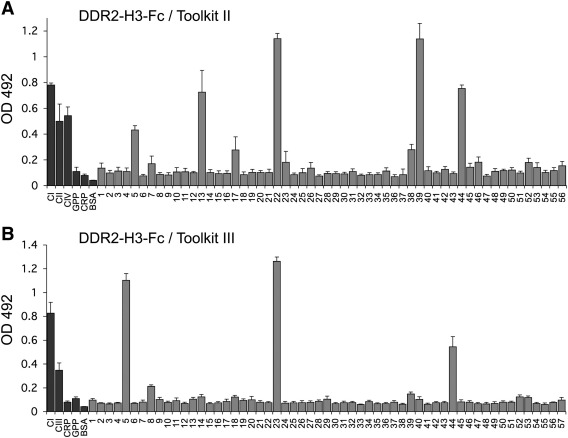
Characterisation of binding sites for the DDR2–DDR1 hybrid protein DDR2-H3 on collagens II and III. Binding of recombinant DDR2-H3-Fc to immobilized collagen II Toolkit peptides (A) or collagen III Toolkit peptides (B) in a solid phase binding assay. Recombinant DDR2-H3-Fc protein was added at 10 μg/ml (110 nM) for 3 h at room temperature to 96 wells coated with collagen or peptides at 10 μg/ml. Bound protein was detected with anti-Fc antibodies and measured as absorbance at 492 nm. Shown are the mean +/− SD of four independent experiments, each performed in triplicates. CI: rat tail collagen I; CII: bovine collagen II; CIII: recombinant human collagen III; CIV: placental collagen IV; GPP and CRP peptides as in Fig. 2.

DDR2-H3-Fc was used to analyse binding sites on collagens II and III, using the Toolkit peptides, as in Figs. 2 and 3. Results with Toolkit II (Fig. 10A) showed a binding pattern that resembled both DDR1 and DDR2, in that peptides, II-5, II-13, II-22, II-39 and II-44 interacted with DDR2-H3, as shown for wild-type DDR2 in Fig. 2B. Binding to peptide II-39 seemed increased compared to wild-type DDR2. Interestingly, however, like DDR1, DDR2-H3 did not bind peptide II-23. The Toolkit III results also showed characteristics of both DDR1 and DDR2 (Fig. 10B). Like wild-type DDR2, DDR2-H3 bound strongly to peptides III-5 and III-23. While peptide III-44 was still recognised, binding seemed to be diminished compared to wild-type DDR2. Like DDR1, DDR2-H3 lacked the ability to bind to III-39.

## 3 Discussion

Here we report a comprehensive analysis of binding sites for DDR1 and DDR2 on the fibrillar collagens II and III. The homologous DDRs both recognised, with precisely the same amino acid requirements, a GVMGFO motif present in the two collagens. Similar to what we reported previously for DDR2 (Konitsiotis et al., 2008), peptides encompassing the GVMGFO motif were able to act as functional DDR1 ligands by inducing receptor autophosphorylation. Apart from GVMGFO-containing peptides, DDR2 bound a greater number of Toolkit peptides than DDR1. However, these additional binding peptides were not able to act as receptor agonists like the GVMGFO-containing peptides. The DDRs differ in their specificity for non-fibrillar collagens. Here we define the molecular basis for the exclusive recognition of the non-fibrillar collagen IV by DDR1: five non-conserved amino acids present on DDR1 loop regions in proximity to the GVMGFO binding pocket are determinants for collagen IV binding.

There is ample evidence that the cartilage-specific collagen II is a physiological ligand for DDR2: DDR2 is expressed in cartilage, is present on chondrocytes, and loss of DDR2 function leads to severe growth defects in humans and mice (Ali et al., 2010; Bargal et al., 2009; Kano et al., 2008; Labrador et al., 2001). Whether collagen II is a physiological ligand for DDR1 is less clear, but DDR1 expression has been reported in chondrocytes in normal articular cartilage (Zeggini et al., 2004). It is thus conceivable that DDR1 might come into contact with collagen II. We previously reported low binding of DDR1 to collagen II, and low level of DDR1 activation by collagen II, compared to collagen II-induced DDR2 activation, concluding that DDR2 rather than DDR1 was a physiological collagen II receptor (Leitinger et al., 2004). Collagen III has been reported to be a ligand for both DDRs (Shrivastava et al., 1997; Vogel et al., 1997). Here we used recombinant human collagen III, to avoid potential contamination with collagen I that is found in many commercial collagen III preparations. We found strong binding of DDR2 to collagen III, with similar signal strength as its binding to collagens I and II (Figs. 2, 3 and data not shown), but the DDR1 ectodomain bound immobilized collagen III poorly (Fig. 3 and data not shown). However, when expressed as full-length receptors in cells, both DDRs could be activated by collagen III added in solution (Supplementary Fig. 4), consistent with collagen III being a ligand for both DDRs.

The Collagen Toolkit peptides have been used in several studies for mapping ligand binding sites on the collagens II and III. Thus, binding sites for von Willebrand factor (VWF) (on collagen III), α2β1 integrin (collagen III), glycoprotein VI (collagen III), and the Ieukocyte associated Ig-like receptors LAIR-1 and LAIR-2 (on collagens II and III) were identified (Jarvis et al., 2008; Lebbink et al., 2009; Lisman et al., 2006; Raynal et al., 2006). With the exception of VWF, whose interaction with collagen III displayed exquisite specificity by binding only a single Collagen III Toolkit peptide, III-23 (Lisman et al., 2006), all other collagen binding proteins bound to several Toolkit peptides. Most of the DDR binding peptides have not been identified as binding to other collagen binding proteins. For example, none of the seven Collagen II Toolkit peptides that bind DDR1 or DDR2 was found to interact with LAIR-1 or LAIR-2 (Lebbink et al., 2009). Moreover, none of the four Collagen III Toolkit peptides that bind DDR2 binds to either glycoprotein VI (Jarvis et al., 2008) or integrin α2β1 (Raynal et al., 2006). Peptides III-5 and III-44, which showed robust binding to DDR2 (Fig. 3), displayed modest binding to LAIR-1 and LAIR-2 (Lebbink et al., 2009).

The DDRs do, however, share an overlapping binding site with other, structurally unrelated collagen binding proteins. The GVMGFO motif present in Toolkit peptides II-22, II-23 and III-23 (and conserved in the α1 chain of collagen I, as discussed previously (Konitsiotis et al., 2008)) is also recognised by VWF (Lisman et al., 2006) and the matricellular protein SPARC (Giudici et al., 2008). While the DDRs bind this motif with an essentially identical collagen binding mode as SPARC (Carafoli et al., 2009; Hohenester et al., 2008), the collagen amino acid requirements for VWF binding are different (Lisman et al., 2006), indicating a distinct binding mode. Recently, the GVMGFO motif was also shown to interact with a mosquito salivary protein, aegyptin (Calvo et al., 2010). The latter interaction, however, differs from the mammalian proteins’ interaction in that it was independent of triple-helical conformation.

The two homologous DDRs share a common overall architecture. Their DS domains are 59% identical in amino acid sequence, with striking conservation of amino acids in the surface-exposed collagen binding loops. The key collagen binding residues in DDR2, delineated by NMR on the isolated DS domain (Ichikawa et al., 2007) as well as by X-ray crystallography of the DDR2 DS domain in complex with a triple-helical peptide encompassing the GVMGFO motif (Carafoli et al., 2009), are all strictly conserved in DDR1 (Fig. 7). These findings are consistent with our data presented here that DDR1 binds the GVMGFO motif with the same collagen amino acid requirements as DDR2 (Figs. 4 and 5). However, despite the conservation of key collagen binding amino acids, we show here that the DDRs differ in their ability to bind Collagen Toolkit peptides. DDR2 displayed robust binding to several peptides on both Collagen II and III Toolkits, while DDR1 binding was restricted mostly to GVMGFO-containing peptides (Figs. 2 and 3). While both DDRs are widely expressed in several organs of the body, a key difference is that DDR1 is mainly found on epithelial cells while DDR2 is found on stromal cells of mesenchymal origin (Alves et al., 1995). Our finding that DDR1 binding to fibrillar collagens is more restricted than that of DDR2 could indicate that DDR1 is mainly a receptor for collagen IV in basement membranes underlying epithelial cell sheets.

Even though DDR1 bound strongly to peptides encompassing the GVMGFO motif present in collagens II and III, we found low binding of DDR1 to full-length collagens II and III. This was not due to poor immobilization of the collagens to the plate, as DDR2 gave strong binding signals to the same collagens. However, it is conceivable that full-length collagens immobilize in such a way that the hydrophobic GVMGFO motif is not or poorly accessible, thus giving low DDR1 binding signals, as DDR1 would bind collagens II and III chiefly through the GVMGFO motif. DDR2, on the other hand, would give strong binding to collagen II and III in this scenario, if its additional binding sites are not masked by plate immobilization.

The DDR2 binding peptide sequences of the Collagen III Toolkit, III-5, III-39 and III-44, are unique to collagen III and not conserved in collagen I (α1 chain) or collagen II, while the DDR2 binding peptides of the Collagen II Toolkit, II-13 and II-44, are conserved in the α1 chain of collagen I, as discussed in our previous study (Konitsiotis et al., 2008). Inspection of Toolkit peptide sequences that interact strongly with DDR2 (II-13, II-44, III-5, III-39 and III-44) does not reveal the presence of obvious candidate motifs analogous to the GVMGFO motif. This, combined with the fact that DDR1 does not bind to these peptides and that these peptides are not able to induce strong DDR2 phosphorylation (Fig. 6), suggests that alternative modes exist for recognition of fibrillar collagen by the DDRs. It is conceivable that the interaction of DDR2 with some of these peptides stabilises an inactive DDR2 conformation. Further studies are required to define the amino acid motifs contained in these peptides and the binding mode.

In addition to their distinct binding specificity towards fibrillar collagen motifs revealed in the present study, a key difference in collagen binding specificity of the DDRs is that only DDR1, but not DDR2, is a receptor for collagen IV (Shrivastava et al., 1997; Vogel et al., 1997). We show here that, as predicted from our structural analysis (Carafoli et al., 2009), five solvent-exposed amino acids present in two loop regions adjacent to, but outside, the DDR collagen (GVMGFO) binding interface are largely responsible for collagen IV binding in DDR1 (Figs. 8 and 9). Replacement of DDR2 amino acids with corresponding DDR1 substitutions in individual loops (L4 or L6, Fig. 5) was not sufficient for inducing collagen IV binding in DDR2, but the combined substitutions of L4 and L6 amino acids resulted in binding of collagen IV to the DDR2-H3 ectodomain and in collagen IV-induced DDR2-H3 receptor autophosphorylation. These results indicate that the contacts of DDR1 with collagen IV involve both L4 and L6 residues.

DDR1 binding motifs in collagen IV are unknown at present. Because collagen IV has 6 different α chains that form 3 distinct heterotrimers, mapping of DDR1 binding sites by peptide libraries is made difficult because of technical challenges involved in the synthesis of the required peptides. Based on the crystal structure of SPARC in complex with a GVMGFO-containing peptide (Hohenester et al., 2008), predictions of putative SPARC binding sites with similarities to the GVMGFO motif were made in the [α1(IV)]_2_α2(IV) heterotrimer (Hohenester et al., 2008). Assuming that DDR1 shares the same binding mode with regards to collagen IV binding, four putative GFO-containing binding sites for DDR1 can be identified (for details see (Hohenester et al., 2008). However, further studies are needed to understand the DDR1–collagen IV interaction in more detail. In addition to its collagen IV-binding ability, the DDR2-H3 construct shared some DDR1 characteristics regarding binding to the Toolkit peptides, suggesting that the specificity for fibrillar collagen motifs other than GVMGFO is determined by DDR amino acids at the periphery of the GVMGFO peptide-binding interface.

In conclusion, our comprehensive analysis using Toolkit peptides has defined binding motifs for both DDRs on the fibrillar collagens II and III. Furthermore, we determined the molecular basis for the specificity of collagen IV recognition by DDR1.

## 4 Experimental procedures

### 4.1 Cell culture and cell lines

Human embryonic kidney (HEK) 293 cells (ATCC, Manassas, VA), HEK293-EBNA cells (Invitrogen) and HEK293-T cells (ATCC) were cultured in Dulbecco's modified Eagle's medium/F12 nutrient mixture (Invitrogen) with 10% fetal bovine serum.

### 4.2 Chemicals and reagents

Bovine serum albumin was obtained from Fisher Scientific, (Loughborough, UK). κ-Casein (From bovine milk; C-0406), collagen I (acid-soluble from rat tail; C-7661), and collagen IV (acid-soluble from placenta; C-5533) were purchased from Sigma (Poole, UK). Bovine collagen II, enzyme-linked immunosorbent assay grade, was from Chondrex Inc. (Redmond, WA). The antibodies and their sources were as follows: goat-anti-DDR2 (AF2538) from R&D Systems (Abingdon, UK); rabbit-anti-DDR1 (SC-532) from Santa Cruz Biotechnology Inc. (Santa Cruz, CA); peroxidase-conjugated goat-anti-human Fc from Jackson ImmunoResearch Laboratories (West Grove, PA); and mouse anti-phosphotyrosine, clone 4G10, from Upstate Biotechnology (Lake Placid, NY). Secondary antibodies were as follows: rabbit-anti-goat Ig-horseradish peroxidase (Zymed Laboratories Inc., San Francisco, CA); goat-anti-rabbit Ig-horseradish peroxidase (P0448, DAKO A/S, Denmark); and sheep-anti-mouse Ig-horseradish peroxidase (Amersham Biosciences UK, Chalfont St Giles, UK).

### 4.3 Peptide synthesis

The sequences of the peptides used in this study are shown in Supplemental Tables 1 and 2. Peptides were synthesized by Fmoc (*N*-(9-fluorenyl)methoxycarbonyl) chemistry as C-terminal amides on TentaGel R RAM resin in an Applied Biosystems Pioneer automated synthesizer and purified as described (Raynal et al., 2006). All peptides were verified by mass spectrometry and shown to adopt triple-helical conformation by polarimetry.

### 4.4 DNA constructs and site-directed mutagenesis

Restriction and modification enzymes were purchased from New England Biolabs or Promega (Southampton, UK). PCR amplification of DDR1 and DDR2 cDNAs was performed with *Pfu* DNA polymerase (Stratagene, Amsterdam, the Netherlands). All PCR-derived sequences were verified by DNA sequencing. DDR2-DDR1 hybrid constructs were generated by strand overlap extension PCR, as described (Leitinger, 2003). DDR2 residues in the selected loop regions were replaced by the corresponding amino acids of DDR1 (loop 4: H110L, I112K; loop 6: H172R, S173V, and N175S) using mutagenic primers that introduced the desired mutations. The resulting hybrid DDR2-H1 contains mutations in loop 4 only; DDR2-H2 contains mutations in loop 6 only; and DDR2-H3 contains mutations in both loops. PCR primers used to generate these mutant constructs can be obtained on request.

For transient expression in HEK293 cells, cDNAs encoding full-length wild-type or mutant DDR2 were subcloned into the mammalian expression vector pcDNA3.1/zeo (Invitrogen). cDNA encoding DDR1a, cloned into the mammalian expression vector pRK5, was a kind gift from Dr Michel Faure (SUGEN inc. San Francisco, CA). cDNA encoding the ectodomain of DDR1 (Asp19-Thr416) and cDNA encoding the ectodomain of wild-type DDR2 and its hybrid mutants (Lys22-Thr398) were obtained by PCR amplification and cloned into a modified pCEP-Pu vector containing a human IgG2 Fc sequence (Hussain et al., 2006). These constructs encoded DDR1-Fc2 and DDR2-Fc, respectively. The resulting proteins contain a three amino acid spacer (Ala-Ala-Ala) between the DDR ectodomains and the IgG2 Fc sequence. The cDNA encoding DDR1-Fc, encompassing the DDR1 ectodomain fused to a human IgG1 Fc sequence has been described elsewhere (Leitinger, 2003).

### 4.5 Production and purification of recombinant proteins

The production and purification of recombinant DDR ectodomain proteins were essentially as previously described (Leitinger, 2003). The Fc-tagged DDR constructs with IgG2 sequence (DDR1-Fc2, DDR2-Fc and Fc-tagged DDR2 hybrid constructs) were isolated from episomally transfected HEK293-EBNA cells. DDR1-Fc, containing human IgG1, was isolated from episomally transfected HEK293-T cells. Proteins were purified by affinity chromatography on HiTrap rProtein A column (17-5079-01, GE Healthcare Biosciences, Uppsala, SW) using an ÄKTA™ Purifier (GE Healthcare Biosciences).

Recombinant human procollagen III was expressed in High Five insect cells by co-infecting cells cultured in suspension at a density of 1 × 10^6^ cells/ml with two recombinant baculoviruses coding for pro-α1(III) chains of type III procollagen (Lamberg et al., 1996) and the α and β subunits of collagen prolyl 4-hydroxylase (a double promoter virus) (Nokelainen et al., 1998) at a ratio of 5:1. l-Ascorbic acid phosphate was added to the culture medium daily to a concentration of 80 μg/ml, and the cells were harvested 72 h after infection. The cells were homogenized in 4 vol of 0.6 M acetic acid, incubated for 2 h, and centrifuged at 10,000 × *g* for 30 min. These and all subsequent steps were performed at 4 °C. The remaining pellet was further extracted with 2 vol of 0.6 M acetic acid for 2 h, centrifuged as above, and the supernatant was combined with that obtained from the previous step. NaCl was added to a final concentration of 3 M, followed after a 1-h incubation by the addition of pepsin to a final concentration of 1 mg/ml. The sample was digested for 18 h, centrifuged as above, and the protein pellet obtained was washed twice with 2 vol of 2 M NaCl, 0.05 M Tris buffer, pH 7.4, for 4 h under stirring, the protein pellet being collected by centrifugation as above. The pellet was dissolved in 1 M NaCl, 0.05 M Tris buffer, pH 7.4, for 18 h, and centrifuged as above. Collagen III was reprecipitated from the supernatant by the addition of NaCl to a final concentration of 2 M, incubation for 3 h, and the pellet was collected by centrifugation as above. The pellet was washed twice with 2 vol of 2 M NaCl, 0.05 M Tris buffer, pH 7.4, for 1 h, and collected by centrifugation at 10,000 × *g* for 1 h. The pellet was dissolved in 2 vol of 10 mM HCl for 18 h, centrifuged as above, and 1 vol of 3 M urea, 0.3 M NaCl, 0.15 M Tris buffer was added to the supernatant, the pH was adjusted to 7.4, and the sample was passed through a HiTrap DEAE FF column (Amersham Biosciences). Collagen III was precipitated from the flow-through by the addition of NaCl and acetic acid to final concentrations of 0.9 M and 0.5 M, respectively, and collected by centrifugation as above. The final collagen III pellet was solubilized in 0.1 M acetic acid and stored at −70 °C.

### 4.6 Solid phase collagen binding assays

The assay was done as previously described (Konitsiotis et al., 2008; Leitinger, 2003). Briefly, collagens or collagen peptides were diluted to 10 μg/ml in 50 mM Tris pH 7.5, 100 mM NaCl or in 0.01 M acetic acid, respectively. They were then coated onto Immulon 2HB 96-well plates (Fisher Scientific) overnight at room temperature. Wells were then blocked for 1 h at room temperature with 1 mg/ml bovine serum albumin (for DDR2 proteins) or with 0.05 mg/ml κ-casein (for DDR1 proteins) in phosphate-buffered saline plus 0.05% Tween-20. Recombinant Fc-tagged DDR proteins, diluted in incubation buffer (0.5 mg/ml bovine serum albumin (for DDR2) or 0.05 mg/ml κ-casein (for DDR1) in phosphate-buffered saline plus 0.05% Tween 20), were added for 3 h at room temperature. Wells were washed six times with incubation buffer afterwards. Bound Fc-tagged DDR proteins were then detected with goat-anti-human Fc coupled to horseradish peroxidase (1:3333 dilution), added for 1 h at room temperature followed by six washes. A color reaction was subsequently performed using *o*-phenylenediamine dihydrochloride (Sigma), added for 3–5 min. The reaction was stopped with 3 M H_2_SO_4_, and plates were read at 492 nm on a 96-well plate reader.

### 4.7 Collagen-induced DDR autophosphorylation

The assay was performed as described before (Leitinger, 2003). Briefly, HEK293 cells in 24-well plates were transfected by calcium phosphate precipitation with the stated DDR expression vectors. 24 h later, the cells were incubated with serum-free medium for 16 h. Cells were then stimulated with collagen I (at 10 μg/ml), collagen III (at 10 μg/ml), collagen IV (at 50 μg/ml), or different collagen peptides (at 100–500 μg/ml) for 90 min, at 37 °C. Cells were lysed in 1% Nonidet P-40, 150 mM NaCl, 50 mM Tris, pH 7.4, 1 mM EDTA, 1 mM phenylmethylsulfonyl fluoride, 50 μg/ml aprotinin, 1 mM sodium orthovanadate, and 5 mM NaF. Aliquots of the lysates were analyzed by SDS-PAGE followed by blotting onto nitrocellulose membranes. The duplicate blots were probed with either anti-phosphotyrosine monoclonal antibody or polyclonal anti-DDR2 (or DDR1) antibodies followed by corresponding horseradish peroxidise-conjugated secondary antibodies. Detection was performed using Enhanced Chemiluminescence Plus (Amersham Biosciences) on an Ettan DIGE Imager (GE Healthcare Biosciences).

## Figures and Tables

**Table 1:**
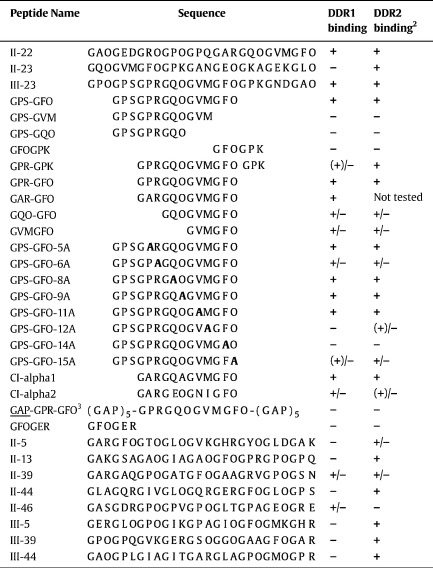
Guest sequences of peptides used to characterise DDR collagen interaction.^a^

^a^*DDR1 binding* denotes binding to DDR1-Fc or DDR1-Fc2; *DDR2 binding* denotes binding to DDR2-Fc or His-DDR2. A plus sign (+) indicates a response similar to peptides II-22, III-23, III-23 or GPS-GFO (control); a minus sign (−) indicates less than 10% of control; (+)/− indicates 10–30% of control; +/− indicates 30–80% of control. ^b^ Data include data from Konitsiotis et al. (2008)*J. Biol. Chem.* 283, 6861–6868. ^c^GAP-GPR-GFO: non-helical peptide flanked by (GAP)_5_ instead of (GPP)_5_ flanking all other peptides.
