# Supplementary material for: Collagen binding specificity of the discoidin domain receptors: Binding sites on collagens II and III and molecular determinants for collagen IV recognition by DDR1
Source: Matrix Biol. 2011 Jan;30(1):16–26. doi: 10.1016/j.matbio.2010.10.004 (PMC3034869; doi:10.1016/j.matbio.2010.10.004)
Supplement: Supplementary file 1 — Supplementary Materials [file mmc1.doc]

**Supplementary Table 1**

**Peptide sequences[[1]](#footnote-2) of Collagen II Toolkit peptides and control peptides**

**1 GPC(GPP)_5_ -GPMGPMGPRGPOGPAGAOGPQGFQGNO-(GPP)_5_GPC-NH_2_**

**2 GPC(GPP)_5_ -GPQGFQGNOGEOGEOGVSGPMGPRGPO-(GPP)_5_GPC-NH_2_**

**3 GPC(GPP)_5_ -GPMGPRGPOGPOGKOGDDGEAGKOGKA-(GPP)_5_GPC-NH_2_**

**4 GPC(GPP)_5_ -GEAGKOGKAGERGPOGPQGARGFOGTO-(GPP)_5_GPC-NH_2_**

**5 GPC(GPP)_5_ -GARGFOGTOGLOGVKGHRGYOGLDGAK-(GPP)_5_GPC-NH_2_**

**6 GPC(GPP)_5_ -GYOGLDGAKGEAGAOGVKGESGSOGEN-(GPP)_5_GPC-NH_2_**

**7 GPC(GPP)_5_ -GESGSOGENGSOGPMGPRGLOGERGRT-(GPP)_5_GPC-NH_2_**

**8 GPC(GPP)_5_ -GLOGERGRTGPAGAAGARGNDGQOGPA-(GPP)_5_GPC-NH_2_**

**9 GPC(GPP)_5_ -GNDGQOGPAGPOGPVGPAGGOGFOGAO-(GPP)_5_GPC-NH_2_**

**10 GPC(GPP)_5_ -GGOGFOGAOGAKGEAGPTGARGPEGAQ-(GPP)_5_GPC-NH_2_**

**11 GPC(GPP)_5_ -GARGPEGAQGPRGEOGTOGSOGPAGAS-(GPP)_5_GPC-NH_2_**

**12 GPC(GPP)_5_ -GSOGPAGASGNOGTDGIOGAKGSAGAO-(GPP)_5_GPC-NH_2_**

**13 GPC(GPP)_5_ -GAKGSAGAOGIAGAOGFOGPRGPOGPQ-(GPP)_5_GPC-NH_2_**

**14 GPC(GPP)_5_ -GPRGPOGPQGATGPLGPKGQTGEOGIA-(GPP)_5_GPC-NH_2_**

**15 GPC(GPP)_5_ -GQTGEOGIAGFKGEQGPKGEOGPAGPQ-(GPP)_5_GPC-NH_2_**

**16 GPC(GPP)_5_ -GEOGPAGPQGAOGPAGEEGKRGARGEO-(GPP)_5_GPC-NH_2_**

**17 GPC(GPP)_5_ -GKRGARGEOGGVGPIGPOGERGAOGNR-(GPP)_5_GPC-NH_2_**

**18 GPC(GPP)_5_ -GERGAOGNRGFOGQDGLAGPKGAOGER-(GPP)_5_GPC-NH_2_**

**19 GPC(GPP)_5_ -GPKGAOGERGPSGLAGPKGANGDOGRO-(GPP)_5_GPC-NH_2_**

**20 GPC(GPP)_5_ -GANGDOGROGEOGLOGARGLTGROGDA-(GPP)_5_GPC-NH_2_**

**21 GPC(GPP)_5_ -GLTGROGDAGPQGKVGPSGAOGEDGRO-(GPP)_5_GPC-NH_2_**

**22 GPC(GPP)_5_ -GAOGEDGROGPOGPQGARGQOGVMGFO-(GPP)_5_GPC-NH_2_**

**23 GPC(GPP)_5_ -GQOGVMGFOGPKGANGEOGKAGEKGLO-(GPP)_5_GPC-NH_2_**

**24 GPC(GPP)_5_ -GKAGEKGLOGAOGLRGLOGKDGETGAA-(GPP)_5_GPC-NH_2_**

**25 GPC(GPP)_5_ -GKDGETGAAGPOGPAGPAGERGEQGAO-(GPP)_5_GPC-NH_2_**

**26 GPC(GPP)_5_ -GERGEQGAOGPSGFQGLOGPOGPOGEG-(GPP)_5_GPC-NH_2_**

**27 GPC(GPP)_5_ -GPOGPOGEGGKOGDQGVOGEAGAOGLV-(GPP)_5_GPC-NH_2_**

**28 GPC(GPP)_5_ -GEAGAOGLVGPRGERGFOGERGSOGAQ-(GPP)_5_GPC-NH_2_**

**29 GPC(GPP)_5_ -GERGSOGAQGLQGPRGLOGTOGTDGPK-(GPP)_5_GPC-NH_2_**

**30 GPC(GPP)_5_ -GTOGTDGPKGASGPAGPOGAQGPOGLQ-(GPP)_5_GPC-NH_2_**

**31 GPC(GPP)_5_ -GAQGPOGLQGMOGERGAAGIAGPKGDR-(GPP)_5_GPC-NH_2_**

**32 GPC(GPP)_5_ -GIAGPKGDRGDVGEKGPEGAOGKDGGR-(GPP)_5_GPC-NH_2_**

**33 GPC(GPP)_5_ -GAOGKDGGRGLTGPIGPOGPAGANGEK-(GPP)_5_GPC-NH_2_**

**34 GPC(GPP)_5_ -GPAGANGEKGEVGPOGPAGSAGARGAO-(GPP)_5_GPC-NH_2_**

**35 GPC(GPP)_5_ -GSAGARGAOGERGETGPOGPAGFAGPO-(GPP)_5_GPC-NH_2_**

**36 GPC(GPP)_5_ -GPAGFAGPOGADGQOGAKGEQGEAGQK-(GPP)_5_GPC-NH_2_**

**37 GPC(GPP)_5_ -GEQGEAGQKGEAGAOGPQGPSGAOGPQ-(GPP)_5_GPC-NH_2_**

**38 GPC(GPP)_5_ -GPSGAOGPQGPTGVTGPKGARGAQGPO-(GPP)_5_GPC-NH_2_**

**39 GPC(GPP)_5_ -GARGAQGPOGATGFOGAAGRVGPOGSN-(GPP)_5_GPC-NH_2_**

**40 GPC(GPP)_5_ -GRVGPOGSNGNOGPOGPOGPSGKDGPK-(GPP)_5_GPC-NH_2_**

**41 GPC(GPP)_5_ -GPSGKDGPKGARGDSGPOGRAGEOGLQ-(GPP)_5_GPC-NH_2_**

**42 GPC(GPP)_5_ -GRAGEOGLQGPAGPOGEKGEOGDDGPS-(GPP)_5_GPC-NH_2_**

**43 GPC(GPP)_5_ -GEOGDDGPSGAEGPOGPQGLAGQRGIV-(GPP)_5_GPC-NH_2_**

**44 GPC(GPP)_5_ -GLAGQRGIVGLOGQRGERGFOGLOGPS-(GPP)_5_GPC-NH_2_**

**45 GPC(GPP)_5_ -GFOGLOGPSGEOGKQGAOGASGDRGPO-(GPP)_5_GPC-NH_2_**

**46 GPC(GPP)_5_ -GASGDRGPOGPVGPOGLTGPAGEOGRE-(GPP)_5_GPC-NH_2_**

**47 GPC(GPP)_5_ -GPAGEOGREGSOGADGPOGRDGAAGVK-(GPP)_5_GPC-NH_2_**

**48 GPC(GPP)_5_ -GRDGAAGVKGDRGETGAVGAOGAOGPO-(GPP)_5_GPC-NH_2_**

**49 GPC(GPP)_5_ -GAOGAOGPOGSOGPAGPTGKQGDRGEA-(GPP)_5_GPC-NH_2_**

**50 GPC(GPP)_5_ -GKQGDRGEAGAQGPMGPSGPAGARGIQ-(GPP)_5_GPC-NH_2_**

**51 GPC(GPP)_5_ -GPAGARGIQGPQGPRGDKGEAGEOGER-(GPP)_5_GPC-NH_2_**

**52 GPC(GPP)_5_ -GEAGEOGERGLKGHRGFTGLQGLOGPO-(GPP)_5_GPC-NH_2_**

**53 GPC(GPP)_5_ -GLQGLOGPOGPSGDQGASGPAGPSGPR-(GPP)_5_GPC-NH_2_**

**54 GPC(GPP)_5_ -GPAGPSGPRGPOGPVGPSGKDGANGIO-(GPP)_5_GPC-NH_2_**

**55 GPC(GPP)_5_ -GKDGANGIOGPIGPOGPRGRSGETGPA-(GPP)_5_GPC-NH_2_**

**56 GPC(GPP)_5_ -GPRGRSGETGPAGPOGNOGPOGPOGPO-(GPP)_5_GPC-NH_2_**

**GPP GPC(GPP)_10_-GPC-NH_2_**

**CRP GPC(GPO)_10_-GPC-NH_2_**

**Supplementary Table 2**

**Peptide sequences of Collagen III Toolkit peptides**

**1 GPC(GPP)_5_ -GLAGYOGPAGPOGPOGPOGTSGHOGSO-(GPP)_5_GPC-NH_2_**

**2 GPC(GPP)_5_ -GTSGHOGSOGSOGYQGPOGEOGQAGPS-(GPP)_5_GPC-NH_2_**

**3 GPC(GPP)_5_ -GEOGQAGPSGPOGPOGAIGPSGPAGKD-(GPP)_5_GPC-NH_2_**

**4 GPC(GPP)_5_ -GPSGPAGKDGESGROGROGERGLOGPO-(GPP)_5_GPC-NH_2_**

**5 GPC(GPP)_5_ -GERGLOGPOGIKGPAGIOGFOGMKGHR-(GPP)_5_GPC-NH_2_**

**6 GPC(GPP)_5_ -GFOGMKGHRGFDGRNGEKGETGAOGLK-(GPP)_5_GPC-NH_2_**

**7 GPC(GPP)_5_ -GETGAOGLKGENGLOGENGAOGPMGPR-(GPP)_5_GPC-NH_2_**

**8 GPC(GPP)_5_ -GAOGPMGPRGAOGERGROGLOGAAGAR-(GPP)_5_GPC-NH_2_**

**9 GPC(GPP)_5_ -GLOGAAGARGNDGARGSDGQOGPOGPO-(GPP)_5_GPC-NH_2_**

**10 GPC(GPP)_5_ -GQOGPOGPOGTAGFOGSOGAKGEVGPA-(GPP)_5_GPC-NH_2_**

**11 GPC(GPP)_5_ -GAKGEVGPAGSOGSNGAOGQRGEOGPQ-(GPP)_5_GPC-NH_2_**

**12 GPC(GPP)_5_ -GQRGEOGPQGHAGAQGPOGPOGINGSO-(GPP)_5_GPC-NH_2_**

**13 GPC(GPP)_5_ -GPOGINGSOGGKGEMGPAGIOGAOGLM-(GPP)_5_GPC-NH_2_**

**14 GPC(GPP)_5_ -GIOGAOGLMGARGPOGPAGANGAOGLR-(GPP)_5_GPC-NH_2_**

**15 GPC(GPP)_5_ -GANGAOGLRGGAGEOGKNGAKGEOGPR-(GPP)_5_GPC-NH_2_**

**16 GPC(GPP)_5_ -GAKGEOGPRGERGEAGIOGVOGAKGED-(GPP)_5_GPC-NH_2_**

**17 GPC(GPP)_5_ -GVOGAKGEDGKDGSOGEOGANGLOGAA-(GPP)_5_GPC-NH_2_**

**18 GPC(GPP)_5_ -GANGLOGAAGERGAOGFRGPAGPNGIO-(GPP)_5_GPC-NH_2_**

**19 GPC(GPP)_5_ -GPAGPNGIOGEKGPAGERGAOGPAGPR-(GPP)_5_GPC-NH_2_**

**20 GPC(GPP)_5_ -GAOGPAGPRGAAGEOGRDGVOGGOGMR-(GPP)_5_GPC-NH_2_**

**21 GPC(GPP)_5_ -GVOGGOGMRGMOGSOGGOGSDGKOGPO-(GPP)_5_GPC-NH_2_**

**22 GPC(GPP)_5_ -GSDGKOGPOGSQGESGROGPOGPSGPR-(GPP)_5_GPC-NH_2_**

**23 GPC(GPP)_5_ -GPOGPSGPRGQOGVMGFOGPKGNDGAO-(GPP)_5_GPC-NH_2_**

**24 GPC(GPP)_5_ -GPKGNDGAOGKNGERGGOGGOGPQGPO-(GPP)_5_GPC-NH_2_**

**25 GPC(GPP)_5_ -GGOGPQGPOGKNGETGPQGPOGPTGPG-(GPP)_5_GPC-NH_2_**

**26 GPC(GPP)_5_ -GPOGPTGPGGDKGDTGPOGPQGLQGLO-(GPP)_5_GPC-NH_2_**

**27 GPC(GPP)_5_ -GPQGLQGLOGTGGPOGENGKOGEOGPK-(GPP)_5_GPC-NH_2_**

**28 GPC(GPP)_5_ -GKOGEOGPKGDAGAOGAOGGKGDAGAO-(GPP)_5_GPC-NH_2_**

**29 GPC(GPP)_5_ -GGKGDAGAOGERGPOGLAGAOGLRGGA-(GPP)_5_GPC-NH_2_**

**30 GPC(GPP)_5_ -GAOGLRGGAGPOGPEGGKGAAGPOGPO-(GPP)_5_GPC-NH_2_**

**31 GPC(GPP)_5_ -GAAGPOGPOGAAGTOGLQGMOGERGGL-(GPP)_5_GPC-NH_2_**

**32 GPC(GPP)_5_ -GMOGERGGLGSOGPKGDKGEOGGOGAD-(GPP)_5_GPC-NH_2_**

**33 GPC(GPP)_5_ -GEOGGOGADGVOGKDGPRGPTGPIGPO-(GPP)_5_GPC-NH_2_**

**34 GPC(GPP)_5_ -GPTGPIGPOGPAGQOGDKGEGGAOGLO-(GPP)_5_GPC-NH_2_**

**35 GPC(GPP)_5_ -GEGGAOGLOGIAGPRGSOGERGETGPO-(GPP)_5_GPC-NH_2_**

**36 GPC(GPP)_5_ -GERGETGPOGPAGFOGAOGQNGEOGGK-(GPP)_5_GPC-NH_2_**

**37 GPC(GPP)_5_ -GQNGEOGGKGERGAOGEKGEGGPOGVA-(GPP)_5_GPC-NH_2_**

**38 GPC(GPP)_5_ -GEGGPOGVAGPOGGSGPAGPOGPQGVK-(GPP)_5_GPC-NH_2_**

**39 GPC(GPP)_5_ -GPOGPQGVKGERGSOGGOGAAGFOGAR-(GPP)_5_GPC-NH_2_**

**40 GPC(GPP)_5_ -GAAGFOGARGLOGPOGSNGNOGPOGPS-(GPP)_5_GPC-NH_2_**

**41 GPC(GPP)_5_ -GNOGPOGPSGSOGKDGPOGPAGNTGAO-(GPP)_5_GPC-NH_2_**

**42 GPC(GPP)_5_ -GPAGNTGAOGSOGVSGPKGDAGQOGEK-(GPP)_5_GPC-NH_2_**

**43 GPC(GPP)_5_ -GDAGQOGEKGSOGAQGPOGAOGPLGIA-(GPP)_5_GPC-NH_2_**

**44 GPC(GPP)_5_ -GAOGPLGIAGITGARGLAGPOGMOGPR-(GPP)_5_GPC-NH_2_**

**45 GPC(GPP)_5_ -GPOGMOGPRGSOGPQGVKGESGKOGAN-(GPP)_5_GPC-NH_2_**

**46 GPC(GPP)_5_ -GESGKOGANGLSGERGPOGPQGLOGLA-(GPP)_5_GPC-NH_2_**

**47 GPC(GPP)_5_ -GPQGLOGLAGTAGEOGRDGNOGSDGLO-(GPP)_5_GPC-NH_2_**

**48 GPC(GPP)_5_ -GNOGSDGLOGRDGSOGGKGDRGENGSO-(GPP)_5_GPC-NH_2_**

**49 GPC(GPP)_5_ -GDRGENGSOGAOGAOGHOGPOGPVGPA-(GPP)_5_GPC-NH_2_**

**50 GPC(GPP)_5_ -GPOGPVGPAGKSGDRGESGPAGPAGAO-(GPP)_5_GPC-NH_2_**

**51 GPC(GPP)_5_ -GPAGPAGAOGPAGSRGAOGPQGPRGDK-(GPP)_5_GPC-NH_2_**

**52 GPC(GPP)_5_ -GPQGPRGDKGETGERGAAGIKGHRGFO-(GPP)_5_GPC-NH_2_**

**53 GPC(GPP)_5_ -GIKGHRGFOGNOGAOGSOGPAGQQGAI-(GPP)_5_GPC-NH_2_**

**54 GPC(GPP)_5_ -GPAGQQGAIGSOGPAGPRGPVGPSGPO-(GPP)_5_GPC-NH_2_**

**55 GPC(GPP)_5_ -GPVGPSGPOGKDGTSGHOGPIGPOGPR-(GPP)_5_GPC-NH_2_**

**56 GPC(GPP)_5_ -GPIGPOGPRGNRGERGSEGSOGHOGQO-(GPP)_5_GPC-NH_2_**

**57 GPC(GPP)_5_ -GERGSEGSOGHOGQOGPOGPOGAOGPC-(GPP)_5_GPC-NH_2_**


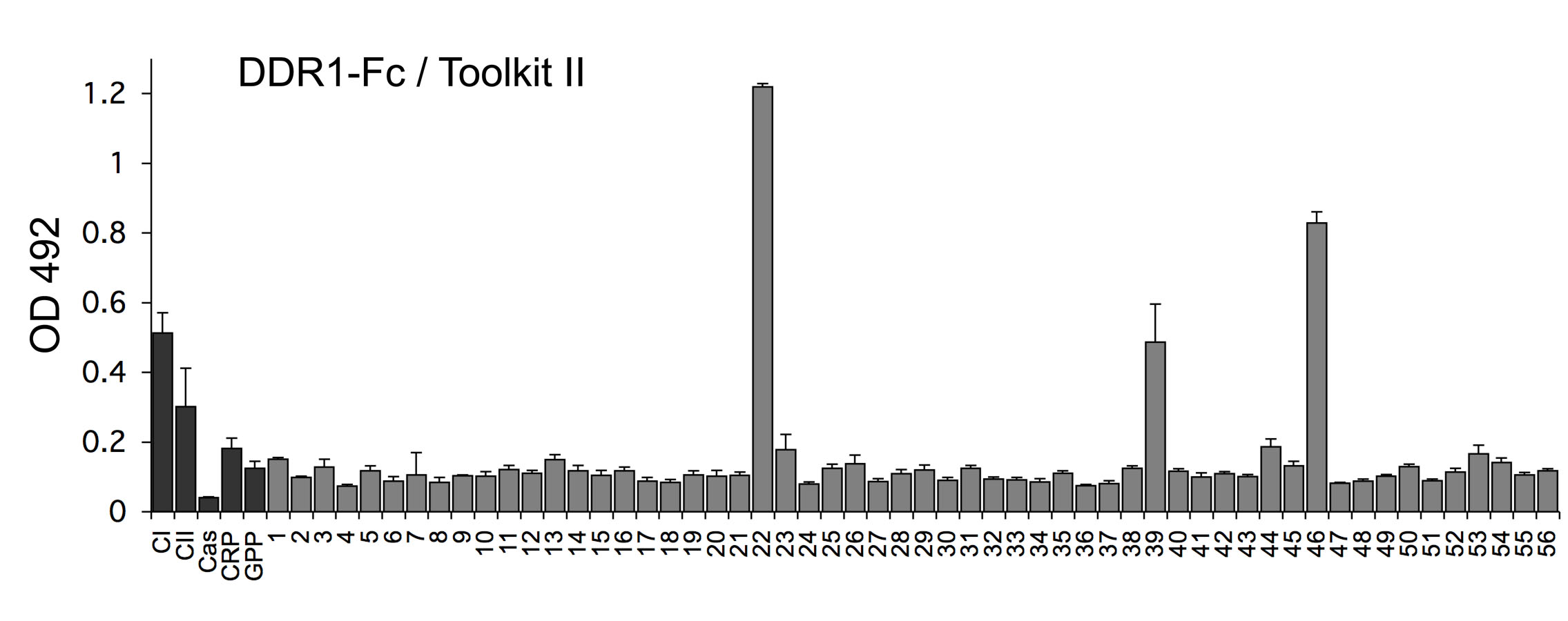


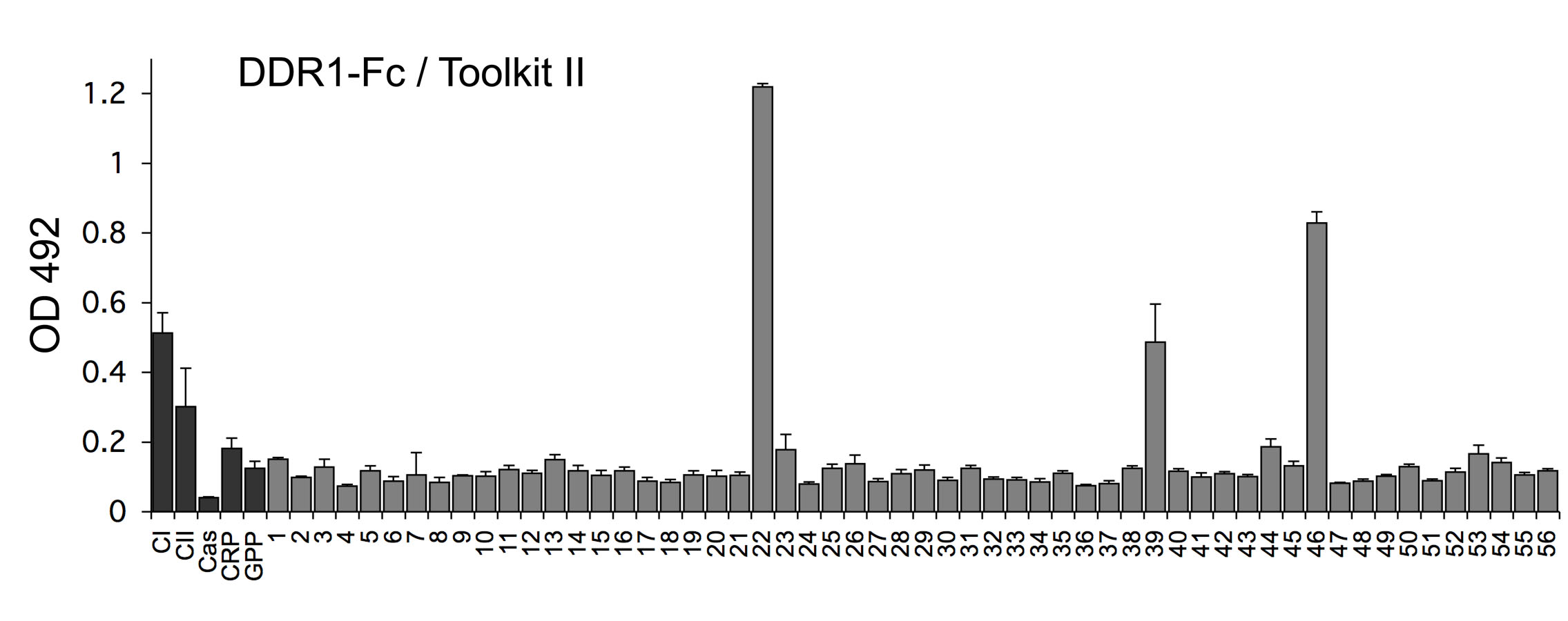
**Supplementary Figure 1.** Identification of DDR1 binding sites on collagen II. Binding of recombinant DDR1-Fc to immobilized collagen II Toolkit peptides in a solid phase binding assay. Recombinant DDR1-Fc was added at 20 μg/ml (220 nM) for three hours at room temperature to 96 wells coated with collagen or peptides at 10 μg/ml. Bound protein was detected with anti-Fc antibodies and measured as absorbance at 492 nm. Shown are the mean +/- SD of four independent experiments, each performed in triplicates. *CI,* rat tail collagen I; *CII*, bovine collagen II; *Cas,* casein; *GPP* and *CRP* peptides as in Figure 2.


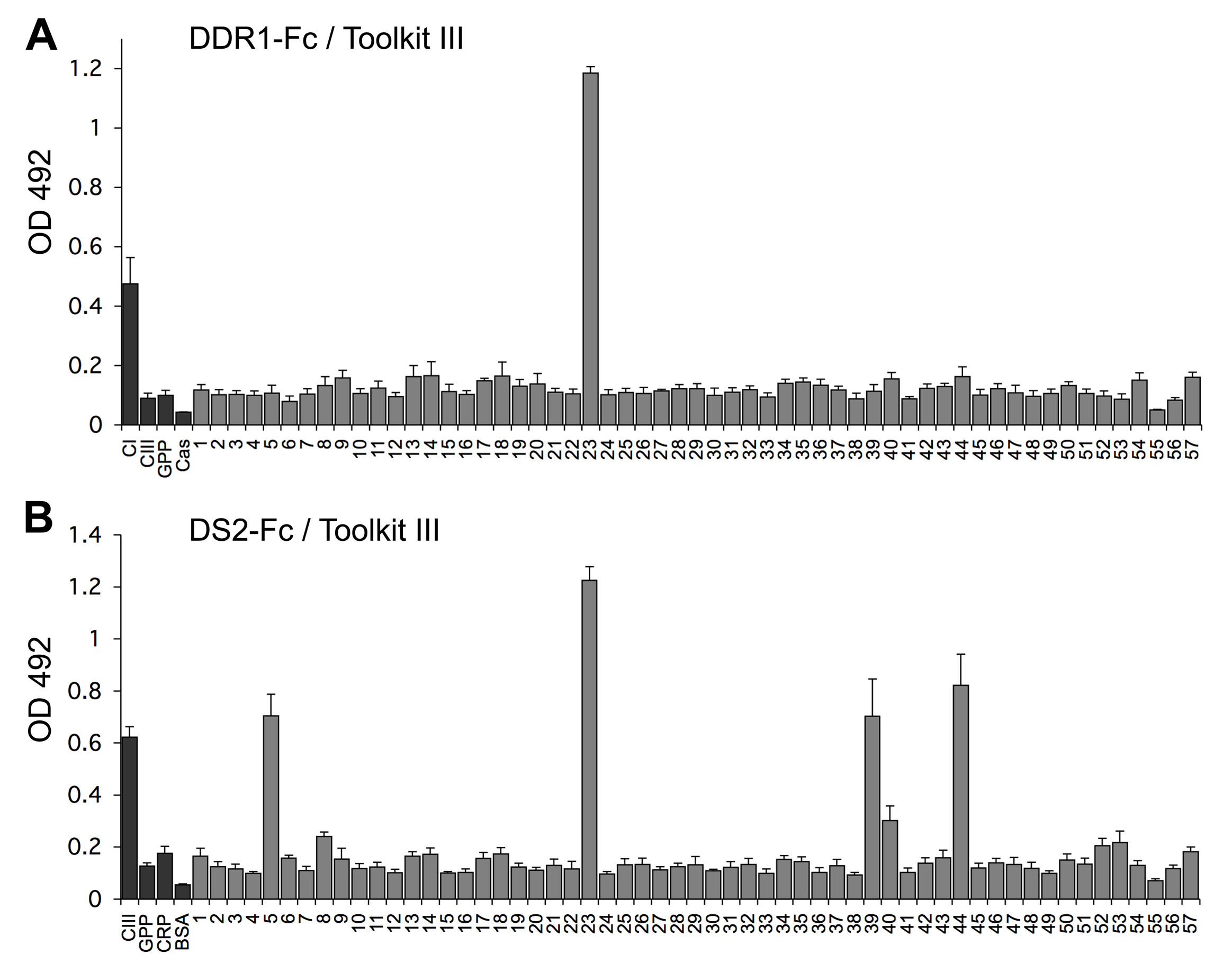


**Supplementary Figure 2.** Identification of DDR1 and DDR2 binding sites on collagen III. Binding of recombinant DDR1-Fc or DS2-Fc to immobilized collagen III Toolkit peptides in a solid phase binding assay. Recombinant DDR-Fc proteins were added for three hours at room temperature to 96 wells coated with collagen or peptides at 10 μg/ml. Bound proteins were detected with anti-Fc antibodies and measured as absorbance at 492 nm. (A) Binding of DDR1-Fc, added at 15 μg/ml (165 nM). (B) Binding of DS2-Fc, added at 10 μg/ml (159 nM). Shown are the mean +/- SD of three to four independent experiments, each performed in triplicates. *CI,* rat tail collagen I; *CIII*, recombinant human collagen III; *Cas,* casein; *GPP* and *CRP* peptides as in Figure 2.


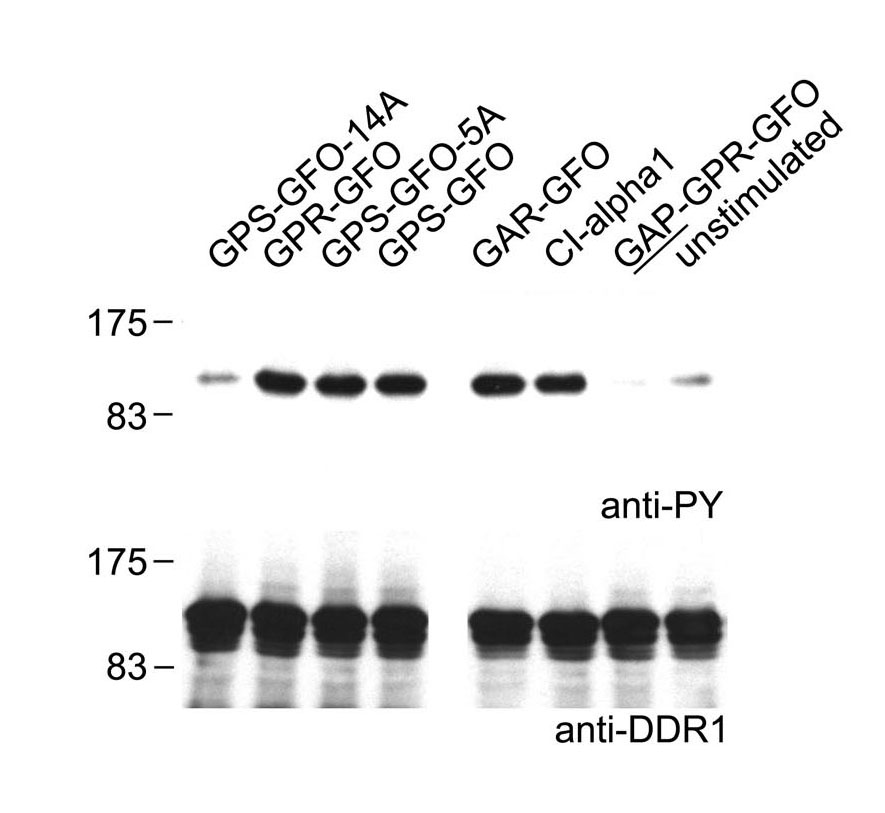


**Supplementary Figure 3.** DDR1 binding peptides mediate autophosphorylation of cell surface DDR1. Full length DDR1a was transiently expressed in HEK293 cells. After stimulation for 90 min with collagen at 10 μg/ml or collagen peptides at 100 μg/ml, cell lysates were analysed by SDS-PAGE and Western blotting. Peptide names refer to Table 1. The blot was probed with anti-phosphotyrosine mAb 4G10 (upper panel), followed by stripping and reprobing with anti-DDR1 (lower panel). The position of molecular weight markers (in kDa) are indicated. The experiment was carried out four times with very similar results.


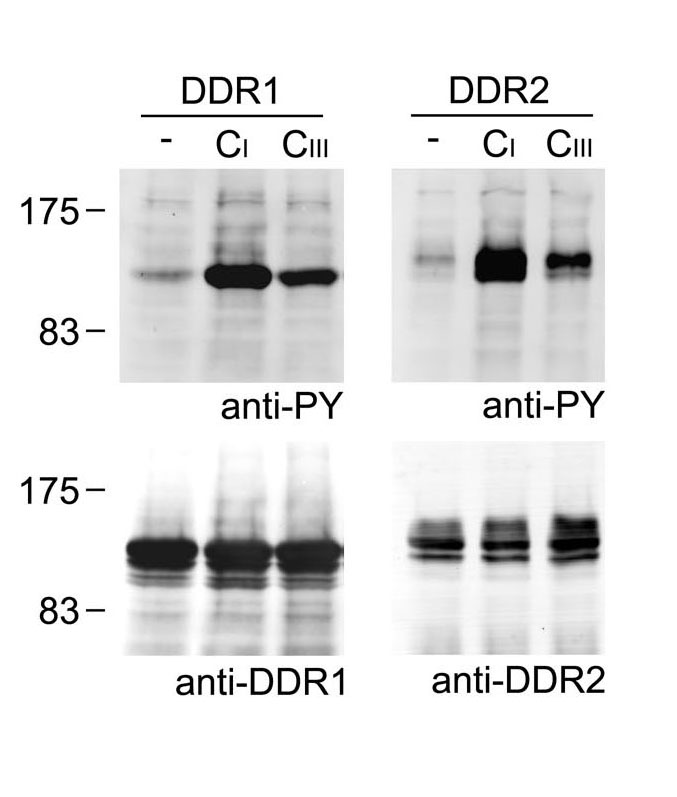


**Supplementary Figure 4.** Collagen III mediates autophosphorylation of DDR1 and DDR2. Full length DDR1a or DDR2 was transiently expressed in HEK293 cells. After stimulation for 90 min with collagen at 10 μg/ml, cell lysates were analysed by SDS-PAGE and Western blotting. For DDR2, cell lysates were resolved on two gels. The corresponding blots were probed with anti-phosphotyrosine mAb 4G10 (upper panel) or anti-DDR2 (lower panel). For DDR1, the blot was probed with anti-phosphotyrosine mAb 4G10 (upper panel), followed by stripping and reprobing with anti-DDR1 (lower panel). The position of molecular weight markers (in kDa) are indicated. *CI,* rat tail collagen I; *CIII,* recombinant human collagen III. The experiment was carried out four times with very similar results.

1. Peptides 1-56 contain amino acid sequences from human collagen II. Peptide #37 could not be synthesised with the wild-type collagen II sequence and a variant, in which Asp^659^ was replaced by Glu (underlined in the table), was therefore synthesised. [↑](#footnote-ref-2)
